# The Anti-Inflammatory Effect of *Lactococcus lactis*-Ling-Zhi 8 on Ameliorating Atherosclerosis and Nonalcoholic Fatty Liver in High-Fat Diet Rabbits

**DOI:** 10.3390/ijms252011278

**Published:** 2024-10-20

**Authors:** Mey-Fann Lee, Nancy M. Wang, Yu-Wen Chu, Chi-Sheng Wu, Wei-Wen Lin

**Affiliations:** 1 Department of Medical Research, Taichung Veterans General Hospital, Taichung 407219, Taiwan; mflee@vghtc.gov.tw (M.-F.L.); luke.chisheng@gmail.com (C.-S.W.); 2 Department of Biology, National Changhua University of Education, Changhua 50007, Taiwan; nancy@cc.ncue.edu.tw; 3 Department of Pharmacy, Taichung Veterans General Hospital, Taichung 407219, Taiwan; yuwenchu@vghtc.gov.tw; 4 Cardiovascular Center, Taichung Veterans General Hospital, Taichung 407219, Taiwan; 5 Department of Post-Baccalaureate Medicine, College of Medicine, National Chung Hsing University, Taichung 402202, Taiwan

**Keywords:** atherosclerosis, nonalcoholic fatty liver disease, Ling-Zhi supplementation, immune dysregulation

## Abstract

Inflammation plays a crucial role in atherosclerosis and nonalcoholic fatty liver disease (NAFLD). We previously engineered a recombinant *Lactococcus lactis* strain expressing the Ling-Zhi immunomodulatory protein (*L. lactis*-LZ8). This study investigated the anti-atherosclerotic effects of *L. lactis*-LZ8 in rabbits fed a high-fat diet (HFD). Changes in body weight, serum lipid profiles, and liver function were monitored. The aorta and liver tissues were analyzed for gross pathology and histopathology. Eight-week administration of *L. lactis*-LZ8 with HFD ameliorated atherosclerosis by downregulating protein and gene expression associated with lipid metabolism and inflammation in the aortas. The rabbits receiving *L. lactis*-LZ8 exhibited a significant dose-dependent reduction in hepatic fat accumulation. RNA sequencing of the livers revealed that inflammatory genes in the *L. lactis*-LZ8 groups were downregulated compared to the HFD group. Disease ontology enrichment analysis indicated that these genes were involved in atherosclerosis. Gene set enrichment analysis plots revealed significant enrichment in the gene sets related to cholesterol homeostasis. CIBERSORT immune cell fraction analysis indicated significant infiltration by regulatory T cells, CD8+ T cells, activated dendritic cells, and natural killer cells in the *L. lactis*-LZ8 group. Our studies underscore LZ8’s role in precision nutrition, providing a potential solution to the current challenges in modifying atherosclerosis and NAFLD.

## 1. Introduction

Cardiovascular diseases (CVDs) continue to be the leading cause of death globally, claiming approximately 17.9 million lives each year [1,2]. Atherosclerosis, a major risk factor for CVDs, involves plaque buildup within artery walls, leading to significant inflammation and narrowing of the arterial lumen [3]. Nonalcoholic fatty liver disease (NAFLD), the most prevalent liver condition, is characterized by fat accumulation exceeding 5% of liver tissue, with an estimated global prevalence of 20–30% [4,5]. Both atherosclerosis and NAFLD are driven by inflammation and are closely associated with the rise in obesity, metabolic syndrome, and type 2 diabetes mellitus [4]. While statins are the most effective lipid-lowering therapy for preventing atherosclerotic CVD (ASCVD) [4,6], there have been no medications to treat fatty liver—until now. In March 2024, the U.S. Food and Drug Administration (FDA) approved a pill named Rezdiffra™, for nonalcoholic steatohepatitis.

The CANTOS clinical trial, which utilized monoclonal antibodies targeting the IL-1β protein, underscored the definitive connection between inflammation and CVD [7,8]. The NLRP3 inflammasome, which is particularly influential in generating the IL-1 family of cytokines, with IL-1β being the most prominent, plays a critical role in inflammation. Emerging treatments include targeted inhibitors of IL-1 isoforms and NLRP3 inflammasome inhibitors, which potentially offer new therapies for a range of CVDs and possibly cancers [9,10]. However, many of these new drugs, currently in phase II to III clinical trials, may require 5–10 years from phase III to U.S. FDA approval. Upon market release, the high cost of these drugs may limit accessibility for the general population. Notably, in the case of the specific IL-1β inhibitor (canakinumab), there was a higher mortality rate due to severe infections in the treatment groups compared to the placebo group (incidence rate, 0.31 vs. 0.18 events per 100 person-years; *p* = 0.02) and a non-significant increase in neutropenia [7]. Therefore, a more affordable and safer medication than the highly specific IL-1β inhibitor is imperative for patients with chronic inflammatory diseases.

Ling-Zhi (*Ganoderma lucidum*), a traditional medicinal mushroom, has been widely used to promote health and longevity [11,12]. Extracts from Ling-Zhi have exhibited effectiveness against COVID-19 infection [13]. Ling-Zhi-8 (LZ8), a protein extracted from the *G. lucidum* fungus, is known for its anti-inflammatory properties [14,15]. LZ8 has been demonstrated to reduce the expression of LPS-induced proinflammatory mediators (IL-6, IL-1β, and CXCL-10) in macrophage cells through the PI3K/Akt and MAPK pathways [16]. This finding aligns with our preliminary data, which indicate that LZ8-containing *Lactococcus lactis* treatment can decrease mRNA levels of IL-1β in a rabbit model of atherosclerosis [17]. Previously, we employed a high-cholesterol diet for 35 days in rabbits, which led to the onset of early atherosclerosis and NAFLD. In the current study, we have modified the rabbit model by incorporating a higher-fat diet and extending the duration to 56 days to induce fully developed atherosclerosis. This study aims to assess the effects of *L. lactis*-LZ8 supplementation on established atherosclerosis and NAFLD in rabbits fed a high-fat diet (HFD).

## 2. Results

### 2.1. Effects of L. lactis-LZ8 Supplementation on Body Weight, Hematological Parameters, and Serum Lipid Profiles in HFD Rabbits

**Figure 1:**
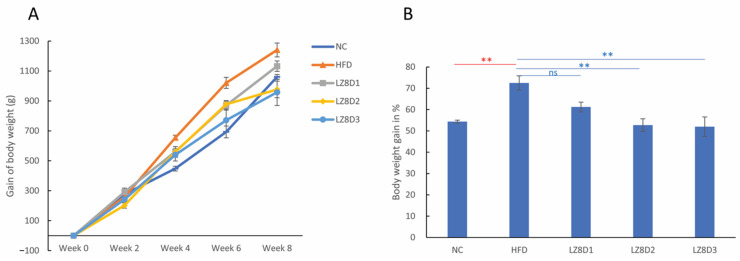
(**A**) Gain in rabbits’ body weight following NC and HFD consumption during 0 to 8 weeks. (**B**) Body weight gain in percentage at week 8. Data expressed as a percentage of body weight gain according to the formula: [(Final wt. − Initial wt.)/Initial wt.] × 100. ** *p* < 0.01; ns, no statistical significance, determined by one-way ANOVA with the Bonferroni multiple range test.

The initial body weights of the 20 rabbits used in the study were 1834 ± 80 g. There was a gradual increase in body weight in either the HFD rabbit group or the normal chow (NC) group during the period from 0 to 8 weeks (Figure 1A). This is consistent with other studies that reported that dietary treatment of rabbits with high-cholesterol diets caused significant weight gain. Interestingly, after *L. lactis*-LZ8 supplementation and HFD for 8 weeks, the gain in the rabbits’ body weight in the LZ8D2 and LZ8D3 groups was significantly lower than that in the HFD group (Figure 1B). *L. lactis*-LZ8 appears to be potentially beneficial for weight management.

**Figure 2:**
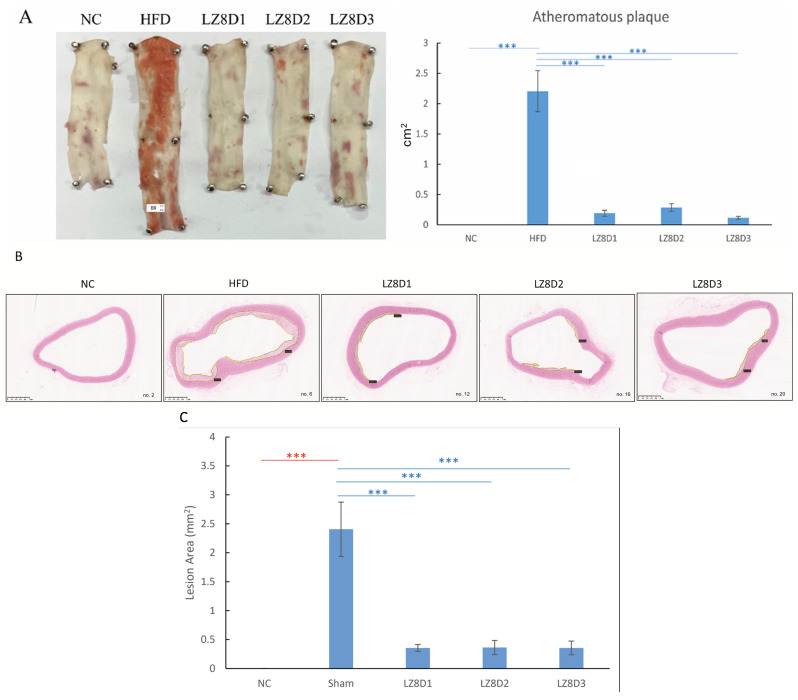
Impact of *L. lactis*-LZ8 supplementation on aortas in experimental rabbit groups. (**A**) Sudan red staining of atheromas in descending aortas and quantity of atheromatous plaques by ImageJ Version 1.54. (**B**) The representative H&E-stained sections of aortic arches, and the atherosclerotic lesions were marked. Magnification: 20×, scale bar: 1 mm. (**C**) Intimal thickness in atherosclerotic lesions from Figure S1 (N = 4). Statistical significance was assessed using the Bonferroni multiple range test. *** *p* < 0.001.

The routine hematological parameters were monitored monthly, and the data from week 8 are presented in Supplementary Table S1. The blood tests for WBC, neutrophils, lymphocytes, monocytes, eosinophils, and basophils were all within the normal range for animals, which suggested that no adverse reactions occurred, even in the high-dose *L. lactis*-LZ8 group. As presented in Table 1, the male rabbits fed a diet enriched with 1% cholesterol and 5% peanut oil for 8 weeks exhibited no significant change in plasma γ-GT, albumin, globulin, total protein, blood urea nitrogen, alkaline phosphatase, creatinine, AST, or ALT when compared to the NC rabbits. However, these rabbits developed hypercholesterolemia, with serum cholesterol levels ranging from 2152.5 ± 596.09 to 2813.25 ± 646.19 mg/dL among all the HFD groups, whereas the serum cholesterol of the rabbits fed the normal chow diet was 52.67 ± 31.47 mg/dL (Table 1). In our model, after 8 weeks of HFD, the blood cholesterol levels increased approximately 40- to 60-fold. We hypothesize that *L. lactis*-LZ8 supplementation might benefit the prevention of atherosclerosis under continuous high-cholesterol conditions. The renal and liver function had no significant change in either the low-dose (LZ8D2) or the high-dose (LZ8D3) group; this result shows that *L. lactis*-LZ8 has a wide range of safety. Low doses of *L. lactis*-LZ8 are still effective, and at doses 10 times higher than the standard dose, it is not only effective (Figure 2) but also does no harm to internal organs.

### 2.2. L. lactis-LZ8 Supplementation Reduces Lipid Plaques of the Aortas in HFD Rabbits

HFD rabbits have been used as an animal model for atherosclerosis research owing to their many resemblances to humans. As illustrated in Figure 2A, Sudan red staining of the descending aortas revealed lipid plaques covering the aorta’s internal surfaces in the HFD group, where atherosclerosis lesions were stained red. Meanwhile, the three groups receiving *L. lactis*-LZ8 supplementation for 8 weeks exhibited remarkable improvement in the atheromatous lesions induced by hypercholesterolemia.

To further assess lipid accumulation, H&E staining was performed on the aorta arches, and images of all 20 rabbits are shown in Supplementary Figure S1. The histological examination of these H&E-stained aorta tissues was consistent with the results obtained from the Sudan red stain. The large lumen of the aortas in the NC group exhibited a smooth contour with intact intima and media. In contrast, atherosclerotic plaques emerged in the aortic wall in the HFD group after an 8-week HFD without supplementation. This confirmed that a total of 1% cholesterol and 5% peanut oil for 8 weeks is sufficient to induce established atherosclerosis in rabbits.

In the representative aorta sections in Figure 2B, the formation of arteriosclerotic plaques was reduced to varying degrees in the three groups (LZ8D1, LZ8D2, and LZ8D3) after receiving oral *L. lactis*-LZ8 probiotics for 8 weeks. The *L. lactis*-LZ8 supplement-treated groups only developed small and thinner plaques at the stained aorta portion. Figure 2C summarizes the intima thickness area of atherosclerotic lesions calculated using NDP.view2 Image software in all the groups. *L. lactis*-LZ8 supplementation shows a positive impact in terms of anti-atherosclerotic effects, not only in blocking early-stage atherosclerosis but also in achieving regression in pre-existing lesions.

### 2.3. L. lactis-LZ8 Supplementation Downregulates Protein and Gene Expression Related to Inflammation in the Aortas of HFD Rabbits

**Figure 3:**
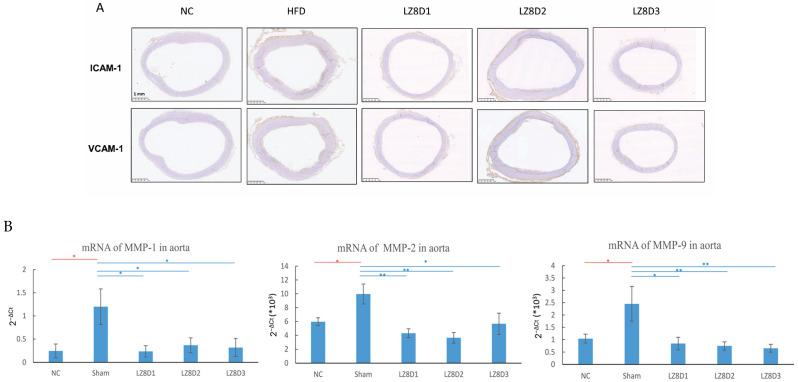
Influence of *L. lactis*-LZ8 supplementation on inflammatory biomarkers in aortas of experimental rabbits. (**A**) Immunohistochemical staining of aortic arch sections, magnification: 20×, scale bar: 1 mm; (**B**) real-time PCR analysis of MMP-1, -2, and -9 mRNA levels in aortas, expressed as mean 2^−ΔCt^ ± SD. Differences were assessed by the Bonferroni multiple range test. * *p* < 0.05; ** *p* < 0.01.

Arterial lesions from both experimental atherosclerosis models and humans show increased expression of inflammatory biomarkers, such as MCP-1, E-selectin, ICAM-1, and VCAM-1 [18,19]. In this study, the expression of ICAM-1 and VCAM-1 in the aorta arches obtained from each rabbit in the five groups was further analyzed immunohistochemically. As depicted in Figure 3A, representative sections from the NC group showed no staining in the lumen of the aortas. Conversely, ICAM-1 and VCAM-1 were strongly expressed along the aorta’s lumen with fatty streaks in the rabbits of the HFD group fed with an HFD for 8 weeks. The three *L. lactis*-LZ8-supplemented groups demonstrated no or weak expression of both adhesion molecules in the lumen of aortas. The data imply that *L. lactis*-LZ8 possesses significant anti-inflammatory abilities that help prevent atherosclerosis induced by an HFD.

Previously, we demonstrated that oral *L. lactis*-LZ8 inhibited the mRNA expression of IL-1β in the aorta and liver, but there was no significant change in rabbit MCP-1 [17]. Matrix metalloproteinases (MMPs) are involved in the immune response and play a crucial role in vascular inflammation, which is strongly associated with atherosclerosis. Many studies have reported that common cardiovascular agents, such as statins, can inhibit the expression or activity of MMP-1, MMP-2, and MMP-9 [20,21]. To further determine whether the LZ8 molecule suppresses the mRNA expression levels of MMPs, real-time PCR analysis was performed on rabbit ascending aortas, as indicated in Figure 3B. The mRNA levels of MMP-1, -2, and -9 significantly increased in the HFD group compared to that of the NC group. Importantly, our data confirm that oral *L. lactis*-LZ8 exerts an anti-inflammatory effect on gene expression at the transcriptional level of MMPs in aortas, which may lead to improved outcomes in atherosclerosis.

### 2.4. L. lactis-LZ8 Supplementation Reduces Hepatic Lipid Accumulation in HFD Rabbits

**Figure 4:**
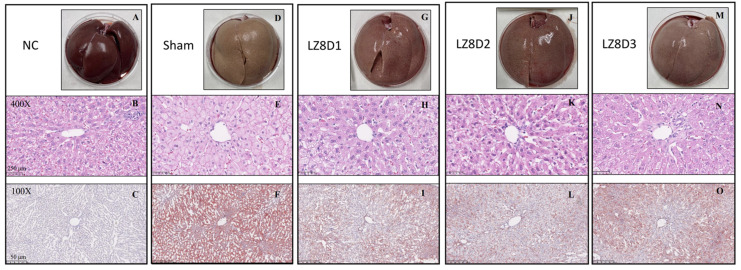
Effects of *L. lactis*-LZ8 on liver tissues in experimental rabbits. Displayed are fresh (**A**,**D**,**G**,**J**,**M**), H&E-stained (**B**,**E**,**H**,**K**,**N**), and Oil Red O-stained (**C**,**F**,**I**,**L**,**O**) liver sections. Magnification and scale bars as indicated.

Clinical studies have demonstrated a high prevalence of CVD in patients with systemic inflammatory diseases and liver abnormalities [22,23]. Thus, we further examined the anti-inflammatory effects of *L. lactis*-LZ8 in a more serious form of NAFLD induced by an 8-week HFD. Figure 4 illustrates the histomorphology of liver specimens using different staining methods. The images show the typical macroscopic features of a normal liver (Figure 4A–C) and an 8-week HFD-induced steatotic liver (Figure 4D–F) using H&E and Oil Red O staining in the rabbits from the NC and HFD groups, respectively. Remarkably, there was a significant decrease in fat deposition and an improvement in fatty livers across all the groups (low-dose, standard, and high-dose groups) receiving oral *L. lactis*-LZ8 (Figure 4G–O).

### 2.5. RNA Sequence Data of Livers from Rabbits of HFD and LZ8D1 Groups

To provide further insights into the molecular mechanisms involved in the activity of *L. lactis*-LZ8 in rabbit livers, RNA transcripts from two liver samples in each group were sequenced. Supplementary Figure S2A (PCA) and Figure S2B (hierarchical clustering analysis) confirm that the two groups of HFD and LZ8 appear in different gene expressions. Figure S2C shows nine relative genes (ABCA1, SREBF2, MMP2, MMP9, IL-1β, IL-10, CXCL8, SELE, and TNF), including those in the inflammatory response that were downregulated in the LZ8 group compared to the HFD group in the RNA-seq results.

**Figure 5:**
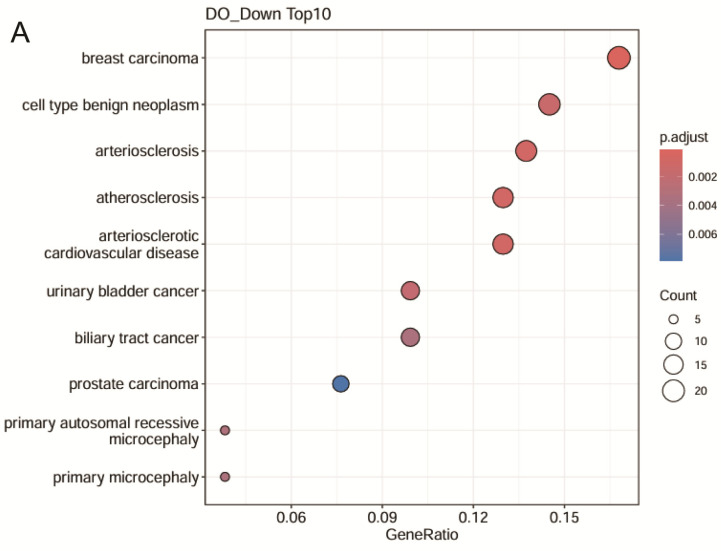

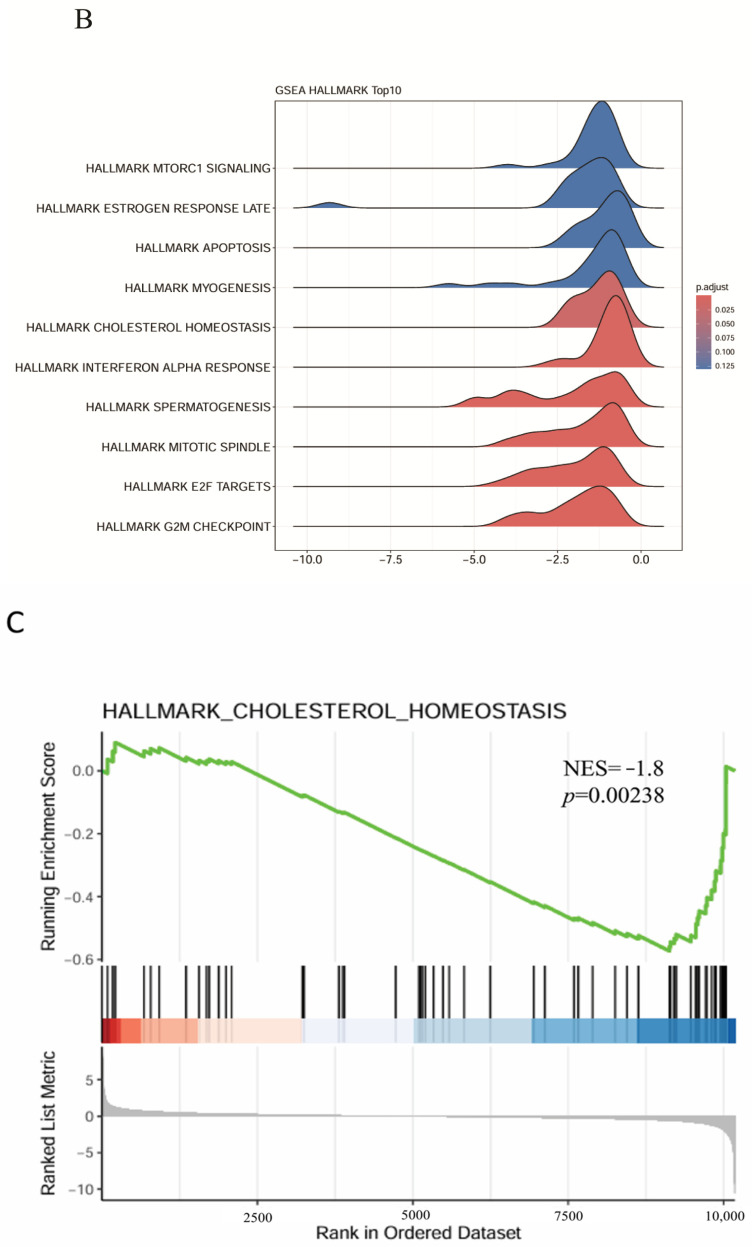
Disease ontology (DO) and gene set enrichment analysis (GSEA) outcomes. (**A**) Top 10 DO terms for downregulated differentially expressed genes (DEGs) with log2FC ≥ 2, color-coded by *p*-value, dot size represents gene counts; (**B**) top 10 statistically significant downregulated pathways in Hallmark GSEA; (**C**) GSEA enrichment plots for Hallmark cholesterol homeostasis, with enrichment score (ES, green line) and indicated normalized enrichment scores (NES) and *p*-values.

A total of 806 DEGs were screened for downregulation using DO enrichment. Among them, there were 201 with statistical significance, and the top 10 diseases are shown in Figure 5A. Interestingly, the DEGs that were discovered were significantly relevant to atherosclerosis and arteriosclerotic CVD and mostly matched the HFD rabbit model, according to the analyses. Table 2 indicates 17 or 18 out of 131 related genes that were associated with the atherosclerosis term in this study. Figure 5B displays the GSEA Hallmark’s top 10 most statistically significant downregulated pathways. The GSEA strategy is a method to identify coordinate changes in gene expression with relevance to biological functions. As expected, GSEA supported the proposal that the gene sets involved in the “cholesterol homeostasis” are a key pathological hallmark in hypercholesterolemia rabbits targeted by LZ8 compared with the HFD control. Figure 5C depicts the magnitude of the log2 FCs for each gene involved in “Hallmark cholesterol homeostasis”, with the enrichment score peaking at the green dotted line. It suggests the downregulation of this pathway (NES = −1.8 and *p* = 0.00238).

**Figure 6:**
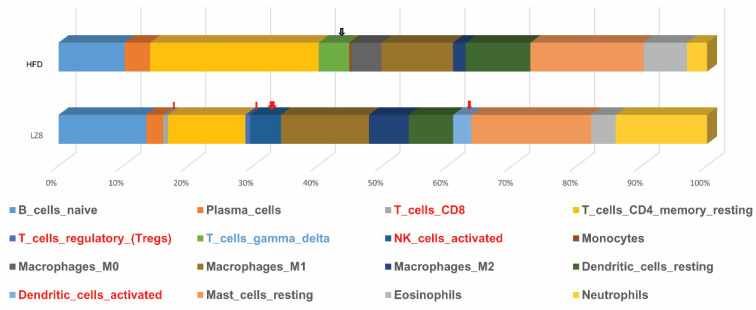
Comparative analysis of immune cell fractions in liver tissues between HFD and LZ8 groups using CIBERSORT.

LZ8 is a protein derived from the fungus *G. lucidum* which has been reported to have immunomodulatory abilities. In the two paired liver samples analyzed by CIBERSORT, the LZ8 group was found to have special infiltration by CD8 T cells, regulatory T cells, activated natural killer cells, and dendritic cells compared to the HFD group (Figure 6). However, in the HFD group, the ratio of gamma delta T cells was increased.

## 3. Discussion

*L. lactis* has long been used in food fermentation and its safety for human consumption makes it an organism that is generally recognized as safe (GRAS). The combination of *L. lactis* NZ3900/pNZ8149 is a widely used system for heterologous protein expression, particularly in food biotechnology [24,25]. *L. lactis* strain NZ3900 is a non-pathogenic and non-colonizing bacterium. Its survival in the gastrointestinal tract typically lasts only 2 to 3 days, and it is usually cleared from the system without colonization of mucosal surfaces [26]. Furthermore, vector pNZ8149 possesses the lacF gene as a food-grade selection marker, which can reduce any antibiotic resistance challenges [27]. The pNZ8149 plasmid, often used with NZ3900, does not carry antibiotic resistance markers, making it suitable for food-related applications. The nisin-controlled gene expression (NICE) system, often associated with pNZ8149, allows the tightly regulated expression of the target protein, which is advantageous for producing proteins that might otherwise be toxic to the host cells [28]. *L. lactis* has emerged over the past two decades as an efficient cell factory for protein expression [29]. On the other hand, LZ8 is a relatively stable protein when exposed to heat, freezing, acidity, and dehydration [30,31]. Therefore, *L. lactis*-LZ8 may be considered a potentially useful additive in food processing and nutritional supplements. Controlled human intervention trials of *Ganoderma lucidum* (Ling-Zhi) have demonstrated its effects on various systems, such as the cardiovascular system [32] and liver [33]. LZ8 expressed in *L. lactis* can also be taken up as a nutrient after mechanical and chemical digestion [34]. Blood from the villous capillaries brings nutrients from the gastrointestinal tract to the liver.

Recent clinical studies have confirmed that even in young adults with hypercholesterolemia, LDL-C may be deposited in the medial layer of the artery leading to atheroma plaque formation [35,36]. These plaques may not present symptoms in the subclinical stages, but as age increases, they soon lead to various ASCVDs, such as stroke and myocardial infarction. According to the lipid hypothesis, the use of statins to reduce LDL-C for the early prevention of arteriosclerosis is currently the most utilized approach [37]. The role of anti-inflammatory treatment in ASCVD is increasingly recognized. For instance, colchicine, an anti-inflammatory drug, was approved for the first time by the U.S. FDA for ASCVD secondary prevention [38].

Previously, we published that oral administration of *L. lactis*-LZ8 was effective in reducing atheroma in the initiation of atherosclerosis [17] and proved its safety with repeated supplementation for 3 months [39]. In this study, we further demonstrated that *L. lactis*-LZ8 was also effective after plaque formation in a hypercholesterolemia animal model, representing the subclinical stage of ASCVD. Additionally, we reported the wide safety range of *L. lactis*-LZ8. The anti-inflammatory effects were consistent without apparent hepatotoxicity or renal toxicity from a low dose (2.5 × 10^10^ CFU/oral) to higher doses (5 × 10^11^ CFU/ora) (Figure 2, Table 2). This gives it a good chance to become a health food supplement in the future.

Recent studies have emphasized that inflammation is the main cause of atherosclerosis. At the early stages of the inflammatory process in atherosclerosis, stimulation and local expression of ICAM-1 and VCAM-1 attract leukocyte recruitment and migration to inflammatory areas [40]. In our study, *L. lactis*-LZ8 administration suppressed the activation of ICAM-1 and VCAM-1 in the lumen of the aorta arch, achieving the effect of preventing the progression of atheroma. ABCA1 mediates the secretion of intracellular free cholesterol to form nascent high-density lipoprotein and plays a key role in cholesterol homeostasis [41]. The cholesterol efflux pathways may suppress inflammasome activation and atherogenesis; however, defects in cholesterol efflux may be a second hit in the progression of atherosclerosis [42,43]. Our results revealed downregulated transcripts of inflammatory cytokines affected by LZ8, including ABCA1, which suggests that cholesterol efflux might also be impaired in the progression of atherosclerosis.

Different anti-inflammation therapies may reverse the progression of the aorta plaque area and intima–media thickness in cholesterol-fed rabbits [44,45]. Previously, we proved that food-grade LZ8 can effectively inhibit inflammatory reactions and the regression of atheroma, confirming its safety and convenience in use [17,39]. In this study, we further verify the efficacy of this anti-inflammatory effect, not only for IL-1β but also for other associated downstream molecules. Notably, the weight gain in the *L. lactis*-LZ8 feeding group was less than that in the HFD group, suggesting anti-inflammatory effects in reducing weight. This is consistent with previous observations that spore powder of *G. lucidum* improves obesity and the inflammatory processes caused by an HFD in obese mice [46]. In high-cholesterol diet rabbits, LZ8 did not reduce appetite, but body weight increased less in the *L. lactis*-LZ8 feeding group. However, this weight loss effect needs further research to be confirmed.

The empirical dietary inflammatory index is a tool to quantify the overall inflammatory potential of a diet. Serum inflammatory biomarker elevation was linearly correlated with diet inflammatory scores and high ASCVD risk [47]. A vegan diet can lower serum inflammation markers, which may help reduce ASCVD risk [48]. Apart from this, NAFLD is thought to be related to mild inflammation of the liver caused by poor dietary habits [49]. Food-grade LZ8, in a series of studies, can effectively reduce these inflammatory reactions and significant improvement is seen in pathology and fatty liver disease.

NAFLD causes cirrhosis of the liver and arteriosclerosis, which are two major causes of death in humans, but the pathophysiology between NAFLD and ASCVD remains unclear; many animal models have been proposed to study the correlation between these two diseases. In this HFD-induced arteriosclerosis rabbit model, excessive fat intake can lead to insulin resistance and systemic inflammation, leading to NAFLD and ASCVD. Many clinical drug trials (ex. statins) can effectively reduce ASCVD mortality, but most of the drugs have no obvious effect on NAFLD. In our series of studies, we evaluate the anti-inflammation effect of *L. lactis*-LZ8 for the dual targeting of NAFLD and ASCVD.

Recent epidemiological research has confirmed the correlation between the liver and cardiovascular disease [50]. In addition, studies have shown that NAFLD-associated pro-inflammatory status can change the structure of the coronary wall, leading to CAD and increased CVD mortality [51]. Therefore, in the experiment shown in Figure 6, we analyzed liver cells to explore whether atherosclerosis is related to the inflammatory mechanism of the liver–heart axis. In Figure 6, we attempt to determine which cells are responsible for inhibiting lipid accumulation in the liver as the consequence of LZ8 treatment. Previous studies showed that both murine and in vitro studies demonstrated that LZ8 can activate murine macrophages and T lymphocytes; this echoes our observations [12,52]. We assume that LZ8 prevents the accumulation of liver fat and the formation of atherosclerosis by regulating immune dysregulation. However, further studies are required.

There are several limitations in this study. First, although lactic acid bacteria are commonly identified as a probiotic strain, in this study we did not discuss their interaction with other intestinal microbiomes, varying food intakes, and individual gastrointestinal absorption. Second, we confirmed the safety and efficacy of LZ8 in a series of animal studies, but we have not yet evaluated its use as a health food supplement. Third, we did not discuss its interaction with lipid-lowering drugs and liver-protective drugs and its effects under different health conditions in humans. Additionally, there was insufficient evidence for this experiment using RNA-seg data to confirm the direct relationship between liver immune cell changes and atherosclerosis improvement. Further studies will focus on exploring the inflammatory mechanism of the liver–heart axis using immunohistological stains.

In conclusion, the RNAseq analysis gives two important findings. The DO (disease ontology) and GSEA (gene set enrichment analysis) of the DEGs showed that the genes involved in the pathways were related to cholesterol homeostasis and that the cells engaged in the pathways evaluated by CIBERSORT were CD8 T cells, regulatory T cells, activated natural killer cells, and dendritic cells. Based on these two factors, we assume that LZ8 prevents cardiovascular disorders by regulating the immune system. There is a need in the future to investigate the underlying mechanism induced by LZ8 which can alter the immune dysfunction. Our studies underscore LZ8’s role in precision nutrition, especially for ASCVD and NAFLD, providing a potential solution to the current challenges in treating these conditions.

## 4. Materials and Methods

### 4.1. Preparation of Biotherapeutic L. lactis-LZ8

The harvested cell pellets of *L. lactis*-LZ8 were obtained from a bench-top fermenter (Firstek, New Taipei City, Taiwan), as previously described [17].

### 4.2. L. lactis-LZ8 Supplementation Treatment in an Established HFD Rabbit Model

All the animal experiments were approved by the Institutional Animal Care and Use Committee of Taichung Veterans General Hospital (approval number: La-1111880). Twenty male New Zealand white rabbits with body weights of 2.0 ± 0.5 kg were acquired from the Livestock Research Institute in Xinhua District, Tainan, Taiwan. Initially, the rabbits were fed a normal chow for 1 week to adapt, and subsequently, they were randomly assigned to five groups of four animals each (Table 3). In this study, there were two negative controls: the NC group was fed with a normal diet, and the HFD group was an untreated control with a high-fat diet. The sentinel control group (NC) received 50 g/kg/day of standard commercial rabbit chow. The other four groups of HFD rabbits were fed with 94% standard chow supplemented with 1% cholesterol and 5% peanut oil for 8 weeks to induce the atherosclerotic process. All the rabbits were housed individually in cages and had unlimited access to water. Table 2 outlines the pilot dose-finding schedule. To evaluate the dose effects of the oral food supplement *L. lactis*-LZ8, the rabbits were given 3 mL of the prepared 15% fructose syrup, as depicted in LZ8D1, LZ8D2, and LZ8D3, once daily on weekdays for 8 weeks. In a previous study (38), we used a fixed dose (4 × 10^10^ CFU/oral) of *L. lactis*-LZ8 to feed the animals once a day on weekdays for 13 weeks, to confirm the safety of *L. lactis*-LZ8. In this study, we used LZ8D1 (5 × 10^10^ CFU/oral) as the standard dose and LZ8D2 (2.5 × 10^11^ CFU/oral) and LZ8D3 (5 × 10^11^ CFU/oral) as the increasingly high doses, to test the effectiveness at different doses and the safety at high doses. The *L. lactis*-LZ8 product was consumed via its incorporation into foods. During the experiment, the body weights of all the rabbits were recorded monthly. Blood samples were collected from the rabbits at baseline and after the 8-week HFD. The samples were drawn from 16 h fasted rabbits using the marginal ear vein or the central ear artery into serum-separating tubes or spray-dried K2EDTA tubes for routine blood tests. At the study’s conclusion, the rabbits were anesthetized with an intramuscular injection of ketamine (40 mg/kg) and xylazine (5 mg/kg), then euthanized. Immediately after thoracotomy, the aortas were excised and meticulously cleaned of surrounding tissue. The aortas were segmented into three parts: the ascending aorta for RNA extraction, the aortic arch for hematoxylin and eosin (H&E) and immunohistochemical (IHC) staining, and the descending aorta for Sudan red staining, respectively. Additionally, livers were promptly removed and divided into two sections, one preserved in RNA for the subsequent RNA extraction and the other fixed in 10% neutral buffered formalin for histological analysis.

### 4.3. Hematology and Serum Lipids Determination

Hematology parameters were measured monthly using an automatic hematology analyzer, Sysmex XN-1000 (Sysmex Corporation, Kobe, Japan). These parameters included total white blood cell (WBC) count, red blood cell count, hemoglobin (HBG) level, hematocrit level, mean corpuscular volume, mean corpuscular HBG (MCH), MCH concentration, platelet level, and differential WBC count. The serum biochemistry parameters of the fasted rabbits were analyzed with an automatic ADVIA Chemistry XPT system (Siemens Healthineers, Forchheim, Germany), assessing total cholesterol, γ-glutamyl transferase (γ-GT), albumin, globulin, total protein, blood urea nitrogen, alkaline phosphatase, creatinine, aspartate transaminase (AST), and alanine transaminase (ALT).

### 4.4. Sudan Red Staining of Rabbit Aortas

Sudan red staining of the aortic intima was performed to analyze fatty infiltration in the experimental rabbits, following the methodology from our previous study. On day 56, the rabbits were euthanized, and the descending aortas were rinsed with PBS, then immersed in a Sudan IV stain solution (5% (*w*/*v*) Sudan IV in 35% ethanol and 50% acetone) at room temperature for 5 min. The tissues were subsequently placed in 80% ethanol for 3 min and washed under running water. The aortas were then longitudinally cut open, exposing the inner lumen, pinned on a rubber pad with the interior facing upwards, and photographed using a digital camera (SONY ZV-1, Sony Corporation, Tokyo, Japan).

### 4.5. H&E Staining of Rabbit Livers and Aorta Arches

Liver tissue samples and aorta arches from all the rabbit groups were fixed in formalin, embedded in paraffin, and sectioned into 5 μm thick slices. The sections were deparaffinized with xylene and rehydrated in a graded series of ethanol for H&E staining. All the slides were examined using a Hamamatsu NanoZoomer 2.0 HT slide scanner ((Hamamatsu Photonics, Hamamatsu City, Japan) at 400× magnification. The extent of the atherosclerotic lesions was analyzed by measuring the intima thickness area using NanoZoomer Digital Pathology (NDP.view2) image software.

### 4.6. IHC Analysis of Rabbit Aorta Arches

The aorta arches were fixed overnight in 4% paraformaldehyde solution, dehydrated in 20% sucrose water, frozen, and embedded in the optimum cutting temperature (OCT) compound. IHC staining of the aorta arches was performed on OCT sections using primary antibodies targeting rabbit intercellular adhesion molecule-1 (ICAM-1, 100-fold dilution) and vascular adhesion molecule-1 (VCAM-1, 200-fold dilution; Bioss, Beijing, China). The sections were incubated with peroxidase-conjugated goat anti-mouse IgG and visualized with 3,3′-diaminobenzidine using a Leica automatic system (Leica, Newcastle, UK).

### 4.7. Oil Red O Staining of Rabbit Livers

Oil Red O staining was performed to detect lipid accumulation in hepatocytes. The liver tissues were fixed overnight in 4% paraformaldehyde solution, dehydrated in 20% sucrose water, and then frozen and embedded in an OCT compound. In brief, the treated hepatocytes were washed with PBS, fixed with 4% paraformaldehyde for 5 min, and then incubated in a 60% isopropanol solution containing 0.5% Oil Red O for 30 min. Finally, the hepatocytes were observed using NDP.view2 viewing software after being photographed with the Hamamatsu slide scanner.

### 4.8. Real-Time Polymerase Chain Reaction of Rabbit Aortas

Total RNAs were extracted from rabbit ascending aortas using TRIzol reagent (Invitrogen, Carlsbad, CA, USA), and cDNAs were synthesized from 2 μg of total RNA using a SuperScript III kit (Invitrogen). The primer pairs used are listed in Supplementary Table S2. The mRNA levels were detected via real-time polymerase chain reaction (PCR) using SYBR Green/ROX PCR master mix (Life Technologies, Carlsbad, CA, USA) on a StepOnePlus™ system (Applied Biosystems, Foster City, CA, USA). PCR amplification was conducted with an initial 10 min step at 95 °C, followed by 40 cycles of 95 °C for 15 s and 60 °C for 1 min. Gene expression levels were quantified relative to the expression of β-actin using the optimized comparative Ct (2^−ΔΔ^Ct) method.

### 4.9. RNA Sequencing of Rabbit Livers and Bioinformatics Analysis

For the RNA sequencing (RNA-seq) analysis, library construction and sequencing analyses were carried out at the Research Technology Support Facility of BIOTOOLS (New Taipei City, Taiwan). Briefly, 2 μg of total RNA per sample were used as input material for the RNA sample preparation. The library was sequenced on an Illumina NovaSeq6000 platform, and 150 bp paired-end reads were generated for each sample. The raw sequenced reads by CASAVA base calling were stored in FASTQ format and were aligned with the rabbit reference genome (*Oryctolagus cuniculus*) downloaded from NCBI (https://www.ncbi.nlm.nih.gov/datasets/genome/GCF_009806435.1/, accessed on 23 January 2022) using the HISAT2.1.0 software (Shanghai Institutes for Biological Sciences, Shanghai, China, 2021). Principal component analysis (PCA) was carried out on the genes significantly expressed among the groups. The rabbit gene symbols were converted to human gene symbols with the Ensembl Biomart tool (http://www.ensembl.org/index.html/, accessed on 23 January 2022). For translating molecular findings to clinical relevance, differentially expressed genes (DEGs) were analyzed using the disease ontology (DO) database (DOID:4) [53]. Furthermore, gene set enrichment analysis (GSEA) with a cluster profile was performed to identify functional alterations between the HFD and LZ8 groups using the Hallmark gene set from the Molecular Signatures Database (Human MSigDB “v2023.2.Hs” updated October 2023) [54]. In the present study, the DEGs were acquired by setting the following thresholds: log2-based fold change (log2 FC) > 2 and *p*-value < 0.05. To investigate the infiltrating immune cells of each liver sample from the HFD rabbits, the cell-type identification by estimating relative subsets of RNA transcripts (CIBERSORT) tool was used between the HFD and LZ8 groups to obtain the immune cell ratio as a percentage.

### 4.10. Statistical Analysis

All the data are presented as means and standard deviations (mean ± SD). The statistical analysis was conducted with the SPSS version 17.0 software (SPSS Inc., Chicago, IL, USA). One-way ANOVA was used for multiple group comparisons, and the Bonferroni multiple range test was used for post hoc examination. Statistical significance was defined as *p* < 0.05.

## Figures and Tables

**Table 1:** Dose effects of oral *L. lactis*-LZ8 in HFD rabbits on clinical chemistry at week 8.

Groups	NC	HFD	LZ8D1	LZ8D2	LZ8D3
Cholesterol (mg/dL)	52.67 ± 31.47	2152.5 ± 596.09 *	2313.5 ± 653.25 **	2586.75 ± 998 **	2813.25 ± 646.19 **
γ-Glutamyltransferase (IU/L)	11.33 ± 1.15	9.75 ± 3.59	7.75 ± 2.75	12.25 ± 0.96	8.75 ± 1.5
	4.17 ± 0.12	4.13 ± 0.26	4.2 ± 0.27	4.05 ± 0.19	4.18 ± 0.15
Globulin (g/dL)	1.5 ± 0.1	1.83 ± 0.41	2 ± 0.52	2.23 ± 0.55	1.98 ± 0.63
Total protein (g/dL)	5.67 ± 0.21	5.95 ± 0.24	6.2 ± 0.6	6.28 ± 0.69	6.15 ± 0.52
Blood Urea Nitrogen (mg/dL)	20.67 ± 1.53	20 ± 2.58	19.75 ± 2.87	17.5 ± 1.29	21.25 ± 4.11
Alkaline Phosphatase (U/L)	215.5 ± 97.5	199.25 ± 123.79	227.38 ± 129.58	153 ± 113.86	131.75 ± 59.66
Creatinine (mg/dL)	1.27 ± 0.06	1.3 ± 0.16	1.28 ± 0.13	1.18 ± 0.19	1.23 ± 0.17
AST (U/L)	31.33 ± 10.21	36.75 ± 4.57	32.75 ± 10.81	40.5 ± 11.47	58.67 ± 4.03
ALT (U/L)	40 ± 3	25.5 ± 4.2	17.5 ± 5.92 **	22.5 ± 9.95 *	20.33 ± 2.87 **

Values are the mean ± SD (*n* = 4/group), * *p* < 0.05, ** *p* < 0.01 by the Bonferroni multiple range test.

**Table 2:** The disease ontology (DO) genes associated with the atherosclerosis term in this study.

ID	Description	GeneRatio	BgRatio	*p* Value	*p* Adjust	*q* Value	Gene ID	Count
DOID:1936	atherosclerosis	17/131	364/10,312	3.3491 × 10^−6^	0.00083924	0.00066311	PLIN2/APOA1/MMP12/CLU/APCS/S100A9/CD163/LPL/SPP1/ALOX15/SAMD9/CPE/RSAD2/TNFRSF12A/SERPINE1/FGF21/CPT1A	17
DOID:2348	arteriosclerotic cardiovascular disease	17/131	365/10,312	3.4753 × 10^−6^	0.00083924	0.00066311	PLIN2/APOA1/MMP12/CLU/APCS/S100A9/CD163/LPL/SPP1/ALOX15/SAMD9/CPE/RSAD2/TNFRSF12A/SERPINE1/FGF21/CPT1A	17
DOID:2349	arteriosclerosis	18/131	411/10,312	4.165 × 10^−6^	0.00083924	0.00066311	PLIN2/APOA1/MMP12/CLU/APCS/S100A9/CD163/LPL/SPP1/ALOX15/SAMD9/CDH13/CPE/RSAD2/TNFRSF12A/SERPINE1/FGF21/CPT1A	18

**Table 3:** Composition of experimental diets and groupings for oral *L. lactis*-LZ8 administration in high-fat diet (HFD) rabbits.

Group	N	Diets during Days 1–56 (50 g/kg/day)	3 mL/oral during Days 1–56
NC	4	Normal chow (NC)	-
HFD	4	94% NC +1% cholesterol + 5% peanut oil	15% fructose syrup
LZ8D1	4	94% NC +1% cholesterol + 5% peanut oil	*L. lactis*-LZ8 in 15% fructose syrup (5 × 10^10^ CFU/oral)
LZ8D2	4	94% NC +1% cholesterol + 5% peanut oil	*L. lactis*-LZ8 in 15% fructose syrup (2.5 × 10^11^ CFU/oral)
LZ8D3	4	94% NC +1% cholesterol + 5% peanut oil	*L. lactis*-LZ8 in 15% fructose syrup (5 × 10^11^ CFU/oral)

## Data Availability

The data needed to evaluate the conclusions are present in the paper.

## Supplementary Materials

The following supporting information can be downloaded at: https://www.mdpi.com/article/10.3390/ijms252011278/s1.
