# Supplementary material for: The Anti-Inflammatory Effect of Lactococcus lactis-Ling-Zhi 8 on Ameliorating Atherosclerosis and Nonalcoholic Fatty Liver in High-Fat Diet Rabbits
Source: Int J Mol Sci. 2024 Oct 20;25(20):11278. doi: 10.3390/ijms252011278 (PMC11508337; doi:10.3390/ijms252011278)
Supplement: Supplementary file 1 [file ijms-25-11278-s001.zip › ijms-3212983-supplementary.pdf]

Supplementary Materials:

Supplementary Figure S1. H&E-stained sections of aortic arches from 20 experimental rabbits in this study. Magnification: 20×, scale bar: 1 mm.

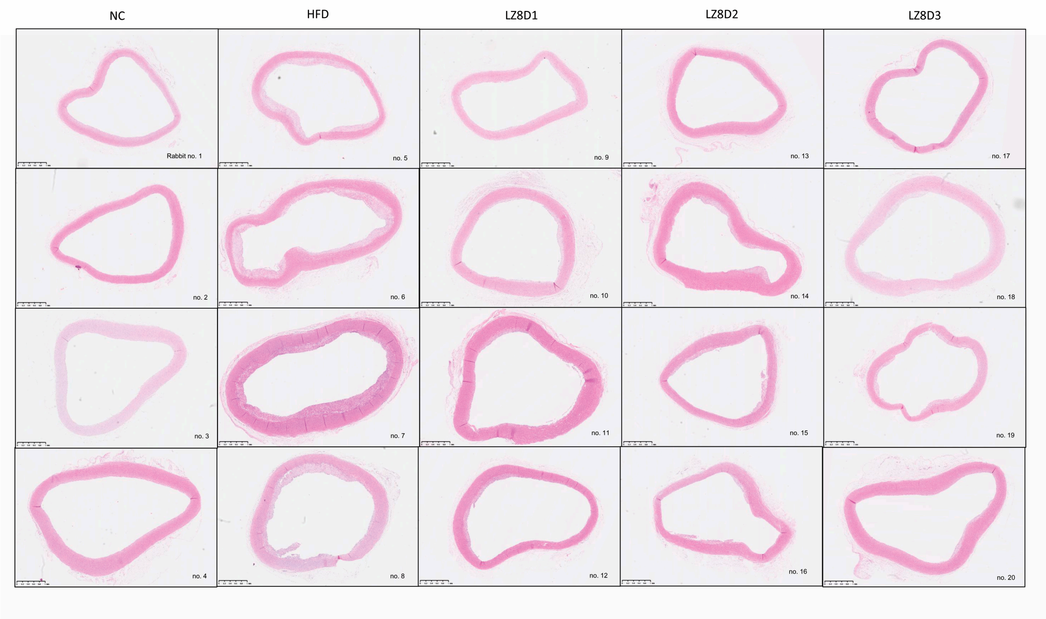

Supplementary Figure S2. RNA-Seq analysis of liver tissues from HFD rabbits. (A) Principle component analysis; (B) Heatmap illustrating hierarchical clustering of hepatocytes from LZ8D1 and HFD groups; (C) Nine downregulated inflammatory cytokine transcripts influenced by LZ8 treatment. Table S1. Dose effects of oral *L. lactis*-LZ8 in HFD rabbits on hematological data at week 8; Table S2. Primer sequences used for real-time PCR.

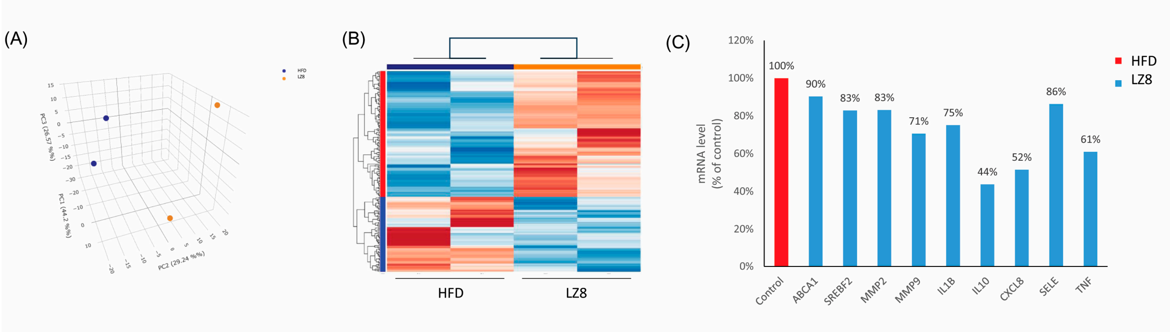

Supplementary Table S1. Dose effects of oral *L.lactis*-LZ8 in HFD rabbits on hematological data at week 8.

Groups	NC	Sham	LZ8D1	LZ8D2	LZ8D3
WBC ($1x10^3/\mu L$)	8.63±2.89	10.95±1.34	9.10±2.95	12.73±2.7	9.83±3.85
RBC ($1x10^6/\mu L$)	5.3±0.1	3.68±0.37	4.02±0.79	3.64±0.62	4.12±0.58
Hb (g/dL)	11.9±0.3	9.58±1.01	10.53±0.22	9.35±1.21	10.08±1.19
HCT (%)	34.91±5.01	27.7±1.51	31.3±2.13	26.65±3.08	30.93±3.2
MCV (fL)	71.8±2.1	81.08±5.85	79.45±11.22	82.53±7.69	75.4±4.19
MCH (pg)	22.77±1.1	25.95±1.59	27.03±6.07	26.33±3.2	24.53±0.77
MCHC (g/dL)	31.67±0.67	32.03±0.59	33.78±2.66	31.88±0.98	32.53±0.95
Platelet ($1x10^3/\mu L$)	265.33±31.47	187.5±53.49	178±56.83	182.25±77.85	150.5±30.56
Neutrophil (%)	19.2±7.3	25.93±6.62	24.2±11.57	34.93±4.96	18.95±9.1
Lymphocyte (%)	74.07±8.19	67.9±5.76	64.85±6.84	59.78±4.8	71.15±3.77
Monocyte (%)	2.93±1.56	4.1±0.61	4.48±0.75	4.03±0.67	3±0.9
Eosinophil (%)	0±0	1.15±2.3	4.08±8.15	0.33±0.65	5.2±5.18
Basophil (%)	3.3±0.44	1.53±1.09	2.4±0.48	1.58±0.98	2.13±0.58

Values are the mean ± SD (n = 4/group)

Supplementary Table S2. Primer sequences used for real-time PCR.

PCR product (size in base pairs)	Forward (5' to 3')	Reverse (5' to 3')
**MMP1** (322)	TCAGTTCGTCCTCACTCCAG	TTGGTCCACCTGTCATCTTC
**MMP2 (**218**)**	GTCTGAAGAGCGTGAAGGTTGG	GTTGACGGGATTGGAGGGGAAG
**MMP9 (**185**)**	CGGAGCACGGAGACGGGTAT	GAAGGGGAAGTGGCAGGGG
**ACTB** (315)	TTCCAGCCCTCCTTCCT	GCCCGACTCGTCATACT
